# Utility of T-cell immunosequencing in distinguishing mycosis fungoides progression from treatment related cutaneous adverse events

**DOI:** 10.3389/fmed.2023.1243459

**Published:** 2023-12-15

**Authors:** Safiyyah Bhatti, Daniel Joffe, Lauren Banner, Sahithi Talasila, Jenna Mandel, Jason Lee, Pierluigi Porcu, Neda Nikbakht

**Affiliations:** ^1^Department of Dermatology and Cutaneous Biology, Thomas Jefferson University, Philadelphia, PA, United States; ^2^Department of Hematology and Oncology, Thomas Jefferson University, Philadelphia, PA, United States

**Keywords:** T-cell immunosequencing, mogamulizumab, mogamulizumab associated rash, mycosis fungoides, mechlorethamine gel

## Abstract

Cutaneous adverse events of both topical and systemic drugs in patients with mycosis fungoides (MF) present a diagnostic challenge as it is often difficult to distinguish drug associated rash from disease progression in the skin. Mogamulizumab and mechlorethamine gel are approved treatments for MF, both of which can cause treatment related cutaneous adverse events. It can often be challenging to distinguish mogamulizumab associated rash (MAR) and mechlorethamine gel associated hypersensitivity dermatitis from MF progression both clinically and histologically. High-throughput sequencing (HTS) of the T-cell receptor (TCR), also known as immunosequencing, can be used to assess T-cell clonality to support a diagnosis of MF. After identification of the malignant TCR clone at baseline, immunosequencing can track the established malignant TCR sequence and its frequency over time with high sensitivity. As a result, immunosequencing clone tracking can aid in distinguishing disease progression from treatment side effects. Here, we present a case series to demonstrate how monitoring of the malignant T-cell frequency by immunosequencing can aid in diagnosis of mogamulizumab and mechlorethamine gel cutaneous adverse events.

## Introduction

Mycosis fungoides (MF) presents a diagnostic challenge to clinicians as it can be difficult to distinguish MF from its clinical and histopathological mimickers, particularly in early stages. Additionally, in patients with MF it can be difficult to distinguish cutaneous eruptions that result from MF treatment from progression of disease. Therefore, an accurate diagnosis of such skin eruptions is crucial in guiding management (1, 2). Two common therapies used in the treatment of MF that are frequently associated with cutaneous side effects include mogamulizumab and mechlorethamine gel. Mogamulizumab is an anti-C-C chemokine receptor 4 monoclonal antibody used in the treatment of refractory MF and Sézary Syndrome (SS) (3). Mechlorethamine gel is a topical nitrogen mustard approved for the treatment of early-stage MF in the United States (4). Both treatments can cause cutaneous side effects in up to 60% of patients that mimic MF clinically and histologically making it challenging to differentiate from MF progression (1, 2).

To make a diagnosis of MF, many factors need to be considered including clinical presentation, histopathological features, immunophenotype, and T-cell clonality (5). Clonality has historically been assessed by polymerase chain reaction-based assays (PCR-electrophoresis). PCR-electrophoresis detects T-cell receptor (TCR)*β* or TCRγ gene rearrangements in tissue samples. This method is unable to identify the exact nucleotide sequences and associated frequencies of the identified TCRs. Instead, it relies on amplicon base-pair length as a proxy for identification of the malignant clone. A normal distribution of amplicons’ base-pair length implies polyclonality; whereas, single peaks suggest monoclonality (6–8).

Immunosequencing, or high-throughput sequencing (HTS) of the TCR has recently emerged as a new and more precise method for assessing T-cell clonality in MF (9, 10). Immunosequencing can identify the precise TCR nucleotide sequences and their frequencies at 100-fold greater sensitivity than PCR-electrophoresis, and can be used in both blood and skin specimens (9–11). Establishment of malignant TCR sequence and frequency using immunosequencing can offer a more robust approach to make an accurate diagnosis when the clinical presentation and histopathology are unclear. Furthermore, after the malignant TCR identity is established at baseline, immunosequencing can aid in distinguishing disease progression from treatment side effect by tracking the established malignant sequence. We present three cases demonstrating how to utilize immunosequencing to distinguish persistent disease from treatment related cutaneous side effects of mogamulizumab and mechlorethamine gel. All patients were consented for participation in this study.

## Case description 1

**Figure 1:**
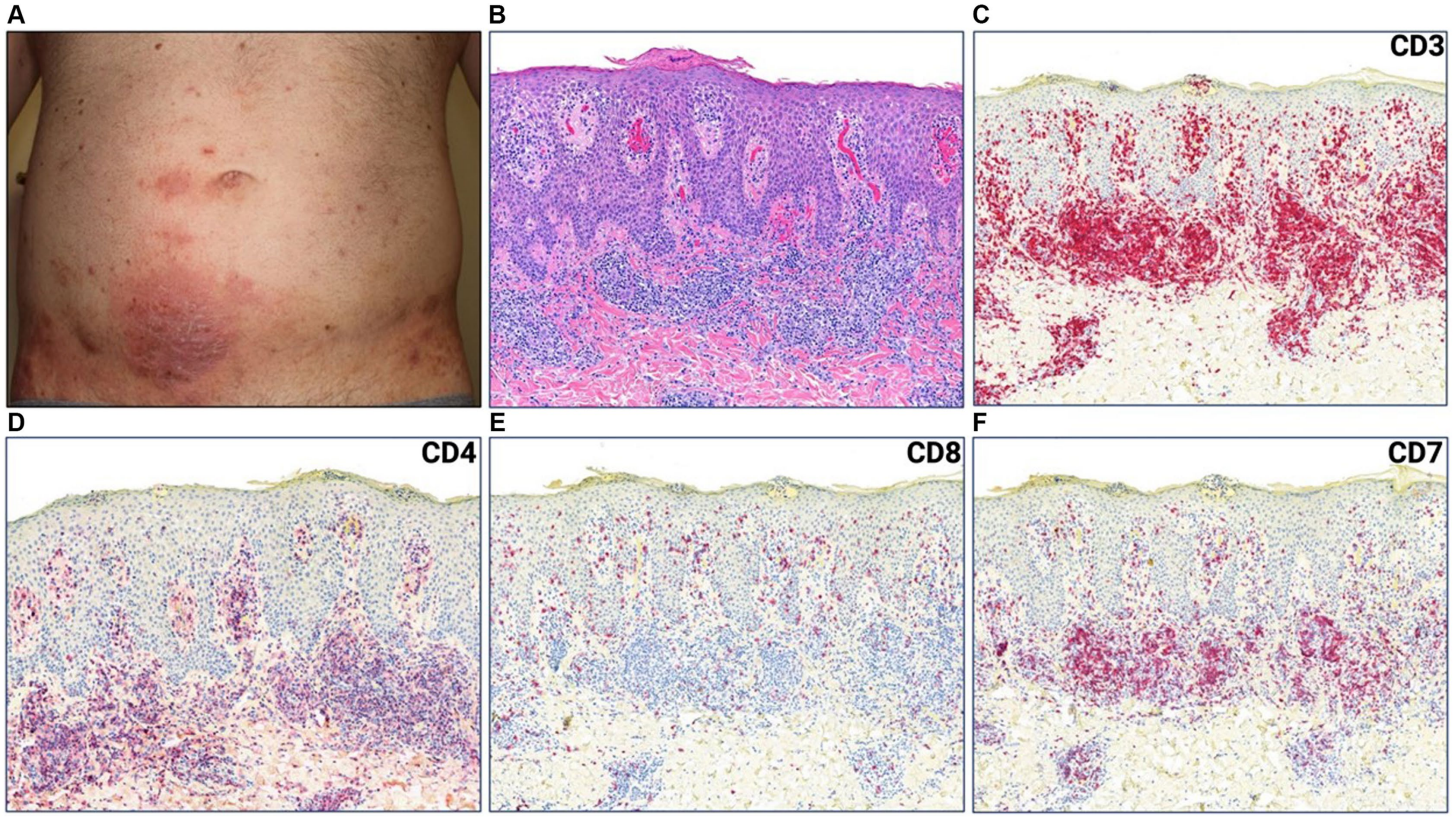
**(A)** Erythematous plaque with scale crust on lower abdomen. **(B)** Punch biopsy demonstrating superficial dermal and intraepidermal infiltrates of atypical lymphocytes with fibroplasia and parakeratosis (H&E 50x). **(C)** CD3, **(D)** CD4, **(E)** CD8, **(F)** CD7 immunostains (H&E 50x).

**Figure 2:**
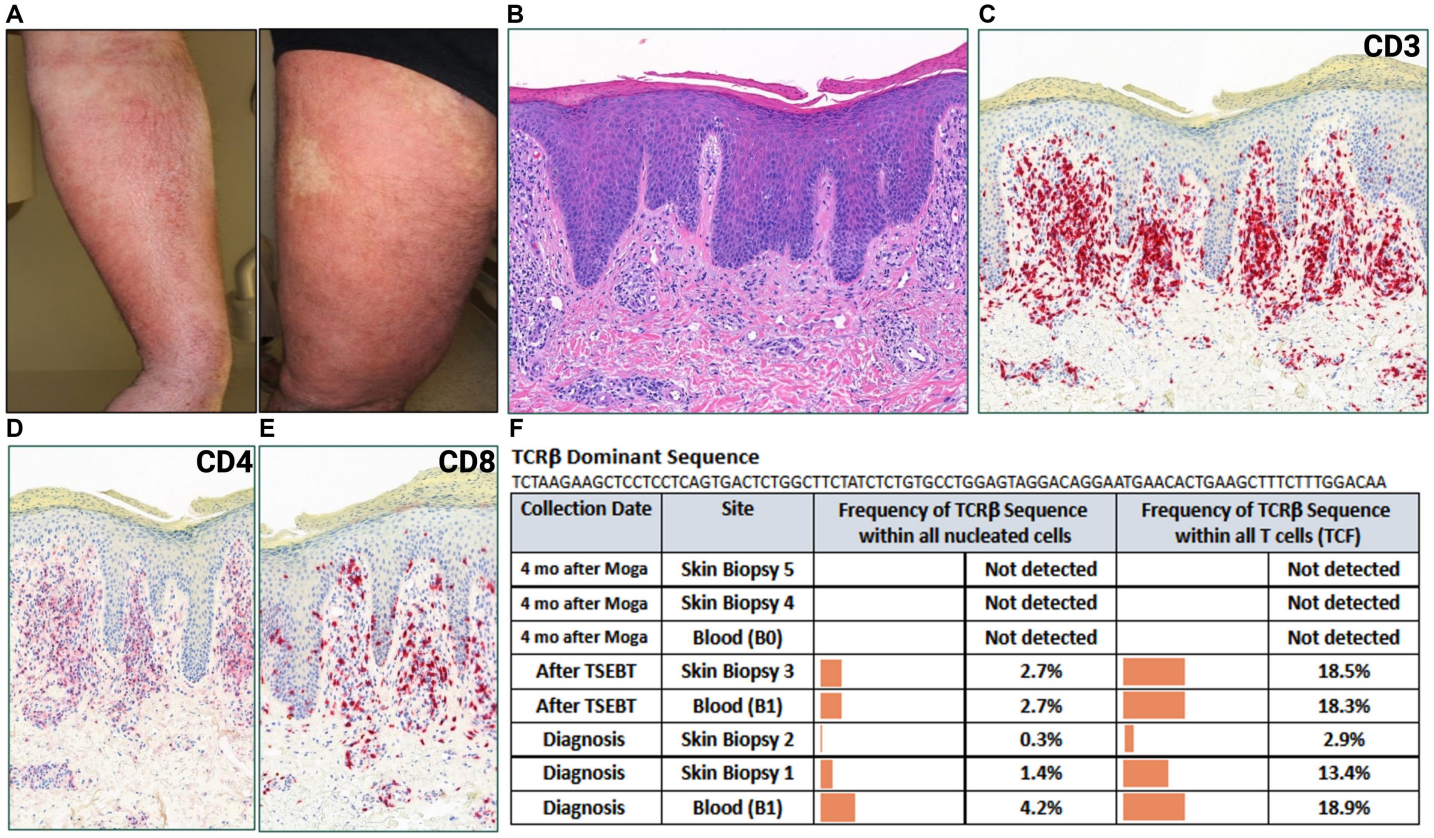
**(A)** Erythematous, lichenified and excoriated plaques with serum seepage on forearm and leg. **(B)** Punch biopsy demonstrating psoriasiform dermatitis with dermal lymphocytic infiltrate (H&E 50x). **(C)** CD3, **(D)** CD4, **(E)** CD8 immunostains (H&E 50x). **(F)** Immunosequencing data demonstrating the TCRβ sequence of the dominant (malignant) clone and its frequency among all cells for each indicated blood or skin biopsy specimen. T-cell Receptor (TCR), Tumor Clone Frequency (TCF), Total Skin Electron Beam Therapy (TSEBT), Moga (Mogamulizumab).

A 44-year-old male with a history of hairy cell leukemia and pediatric gangliocytoma presented with a rash of ten months duration. Physical examination findings are shown in Figure 1A. He underwent biopsy that showed superficial dermal and intraepidermal infiltrates of atypical CD3 expressing lymphocytes with decreased expression of CD7 and slight predominance of CD4 over CD8 (Figures 1B–F). Immunosequencing assay (ClonoSEQ-Adaptive Biotechnologies) identified one dominant TCRβ sequence present in two separate skin biopsies (Figure 2F). Blood immunosequencing also identified a dominant TCRβ sequence identical to the dominant clone detected in skin (Figure 2F). Flow cytometry analysis revealed 600 CD4+/CD26- cells in 1 uL blood indicating B1 status. With 40% body surface area (BSA) involvement and B1 blood status, the patient was diagnosed with stage IB MF. He was initially treated with topical steroids and narrowband ultraviolet B, followed by total skin electron beam therapy and mechlorethamine gel. One year later, blood and skin evaluation revealed the persistence of the dominant clone with sustained frequencies and unchanged B1 status by flow cytometry (Figure 2F). As a result, we began treatment with mogamulizumab infusions.

Four months after starting mogamulizumab therapy, the patient developed a new, worsening rash that clinically resembled his MF (Figure 2A). Biopsy findings demonstrated a psoriasiform spongiotic dermatitis with a dermal infiltrate of CD3+ cells, predominance of CD8 over CD4, and no epidermotropism (Figures 2B–E). Immunosequencing did not detect the previously identified malignant clone or any other dominant clones in either blood or skin biopsies (Figure 2F). Overall findings were consistent with mogamulizumab-associated rash (MAR). He was treated with topical corticosteroids with improvement of the rash and continued mogamulizumab infusions for an additional three months. He achieved complete remission of mycosis fungoides in skin and blood.

## Case description 2

**Figure 3:**
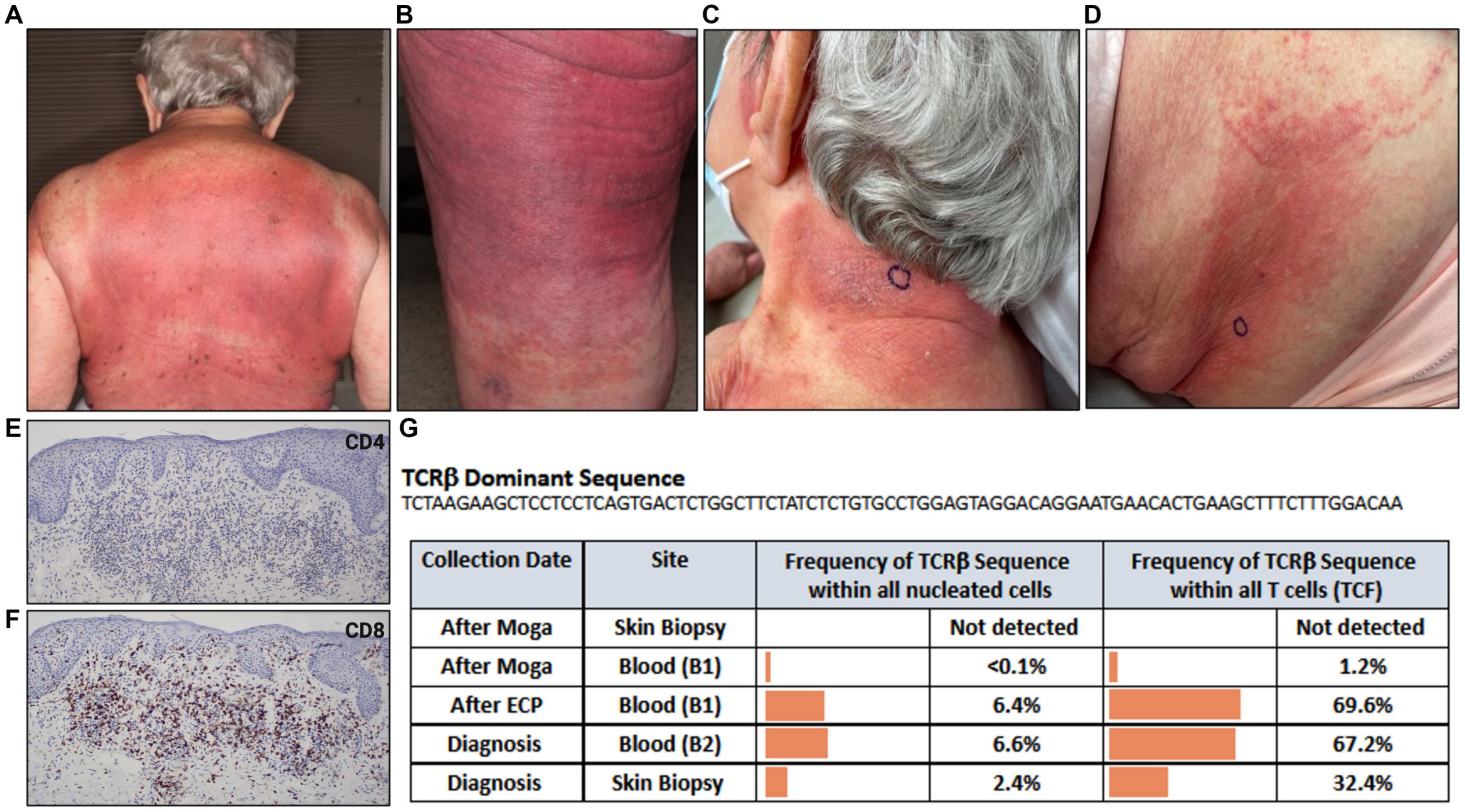
Erythematous patches and thin purple-red plaques involving most skin surface areas on **(A)** back and **(B)** posterior thigh. **(C)** Indurated, lichenified plaque with well-defined border on posterior neck. **(D)** Erythematous papules and ill-defined erythematous patch on flank. **(E)** CD4, **(F)** CD8 immunostains. **(G)** Immunosequencing data demonstrating the TCRβ sequence of the dominant (malignant) clone and its frequency among all cells for each indicated blood or skin biopsy specimen. Extracorporeal Photopheresis (ECP), Moga (Mogamulizumab), T-cell Receptor (TCR), Tumor Clone Frequency (TCF).

An 88-year-old female presented with a pruritic rash on her back, chest, buttocks, and upper and lower extremities (Figures 3A,B). Her BSA was 65% and biopsy of the rash revealed superficial dense lymphocytic infiltrate associated with fibroplasia and epidermotropism. The lymphocytes stained positively for CD3 with decreased expression of CD7, and a predominance of CD4 compared to CD8 especially in the epidermotropic lymphocytes. Immunosequencing identified a dominant TCRβ sequence shared both in the blood and skin (Figure 3G). Peripheral blood flow cytometry analysis revealed 1900 CD4+/CD26- cells in 1 uL blood indicating B2 status. Positron emission tomography/computed tomography (PET/CT) scan showed no metabolically active lymph nodes. Based on skin biopsy results and B2 blood status, the patient was diagnosed with Stage IVA MF. Given a lower level of blood involvement, she was initially treated with extracorporeal photopheresis (ECP), leading to improvement of rash and pruritis. Six months later, restaging of blood revealed B1 status by flow cytometry analysis. In contrast, blood immunosequencing showed unchanged frequencies of the previously identified malignant clone (Figure 3G). Due to the sustained frequencies of the malignant clone in blood, ECP was discontinued, and the patient was treated with mogamulizumab.

Five months after beginning mogamulizumab, the patient developed a new rash on the neck and flank resembling her MF (Figures 3C,D). Biopsy of the flank demonstrated spongiotic dermatitis with a dense superficial infiltrate of mostly lymphocytes. The majority of lymphocytes expressed CD3 and CD8, and a minority expressed CD4 (Figures 3E,F). Peripheral blood flow cytometry analysis detected no evidence of blood involvement (B0 blood status). Immunosequencing did not identify a dominant clone in skin or blood (Figure 3G), excluding MF from the differential and confirming the diagnosis of MAR. She was treated with systemic and topical corticosteroids leading to significant improvement. Subsequently, MAR completely resolved with reduced mogamulizumab infusion frequency (once every 4 weeks). In addition, she achieved complete remission of her mycosis fungoides in both blood and skin.

## Case description 3

**Figure 4:**
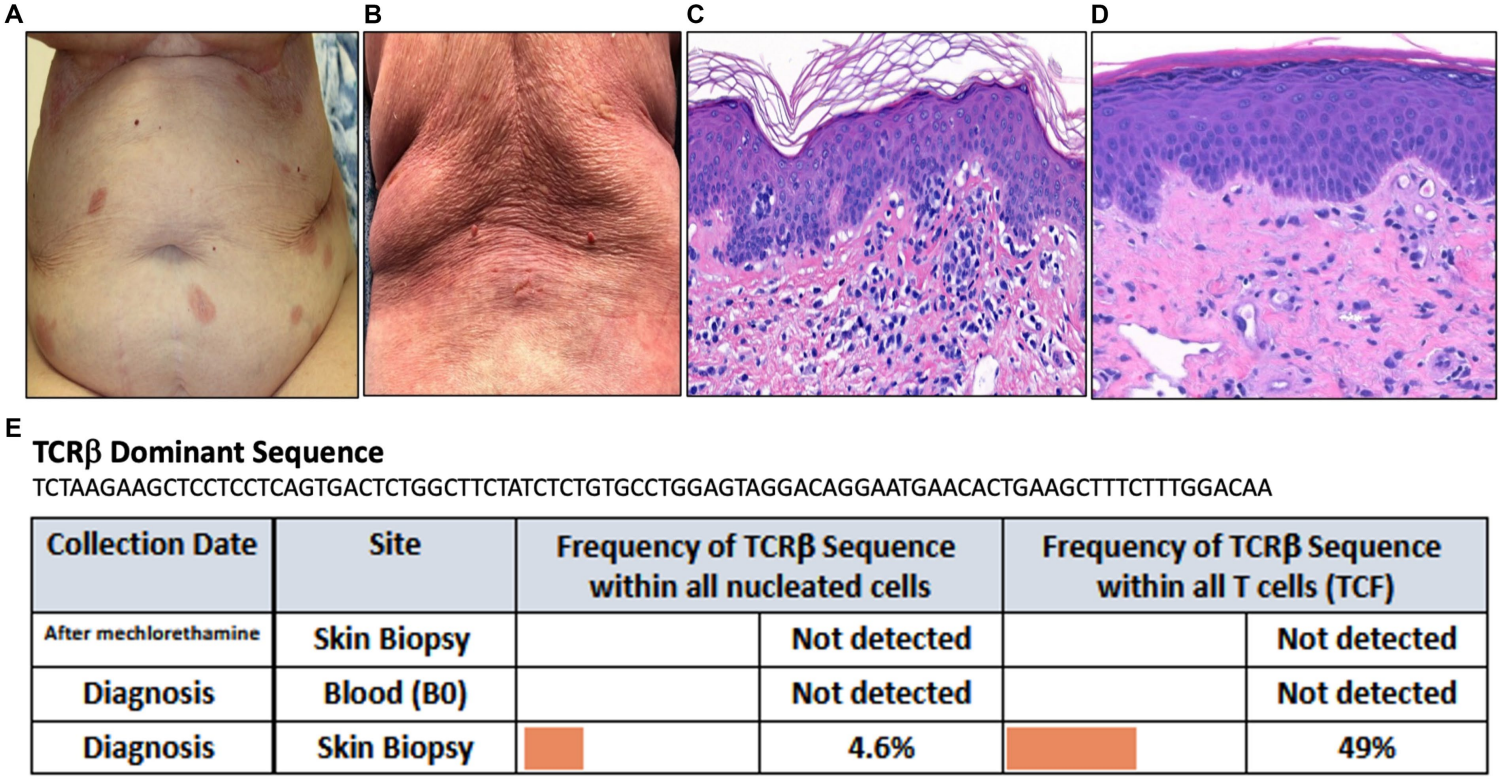
**(A)** Erythematous patches on the abdomen. **(B)** Diffuse erythema on the back. **(C)** Punch biopsy demonstrating superficial dermal fibroplasia with atypical epidermotropic lymphocytes seen in the epidermis as microabscesses (H&E 100x). **(D)** Punch biopsy demonstrating superficial perivascular dermatitis with telangiectasis within the papillary dermis (H&E 100x). **(E)** Immunosequencing data demonstrating the TCRβ sequence of the dominant (malignant) clone and its frequency among all cells for each indicated blood or skin biopsy specimen. T-cell Receptor (TCR), Tumor Clone Frequency (TCF).

A 90-year-old female presented with a year-long history of eczematous patches on the trunk and extremities (Figure 4A). Biopsy of an abdominal lesion demonstrated fibroplasia with dermal and epidermal infiltration of atypical CD3+, CD4+ lymphocytes with diminished CD7 expression that formed Pautrier’s microabscesses (Figure 4C). These immunohistological findings confirmed a diagnosis of MF. Immunosequencing of skin biopsy demonstrated one dominant TCRβ sequence (Figure 4E). Peripheral blood flow cytometry analysis did not detect blood involvement by MF and immunosequencing of the blood did not identify a dominant T-cell clone (Figure 4E).

Mechlorethamine gel treatment was initiated for Stage IA MF. Three months later, she presented to the clinic for an exuberant progressive rash along with a burning sensation across her body with increased pruritus and pain. Physical exam revealed diffuse erythema accompanied by fissuring and oozing in abdominal folds and back (Figure 4B). Given the clinical presentation of extensive skin involvement, there was concern for progression to erythrodermic MF. A biopsy was obtained to distinguish MF progression from mechlorethamine gel associated hypersensitivity dermatitis. The biopsy showed superficial perivascular dermatitis with telangiectasis within the papillary dermis (Figure 4D). Immunosequencing did not reveal a clonal population (Figure 4E). These findings confirmed the diagnosis of cutaneous hypersensitivity reaction secondary to mechlorethamine gel. As a result, mechlorethamine gel was discontinued and the patient received systemic and topical corticosteroid therapy leading to resolution of her rash. Additionally, the patient achieved complete remission of her MF.

## Discussion

In all three cases described, patients developed new rashes after treatment with mogamulizumab or mechlorethamine gel that were clinically similar to their MF presentations. We utilized immunosequencing to monitor response to treatment and to distinguish disease progression from treatment related cutaneous reactions. Immunosequencing initially identified the dominant, presumably malignant, T-cell clone in each case and monitored the frequency of these clones over time. The malignant T-cell clones were not identified by immunosequencing in biopsies obtained from the new skin rashes. This finding along with other histopathological features confirmed the diagnoses of MAR or mechlorethamine gel associated hypersensitivity dermatitis in our cases.

MAR and mechlorethamine gel associated hypersensitivity dermatitis have some distinct histopathological features. MAR may show spongiotic, psoriasiform, interface or granulomatous dermatitis, along with large histiocytes in dermal infiltrate and lack of significant lymphoid atypia (12). Immunohistopathologic findings to help further identify MAR include a decreased or normal CD4:CD8 ratio and retained CD5 and CD7 expression (2). A superficial perivascular dermatitis is the main histopathological finding in mechlorethamine gel associated hypersensitivity dermatitis. Ultimately, TCR sequencing utilizing HTS can also help distinguish MAR and mechlorethamine gel associated hypersensitivity dermatitis from disease progression. In biopsies of MAR or mechlorethamine gel associated hypersensitivity dermatitis, immunosequencing is likely to either not detect the malignant clone or detect its frequency at lower levels that may not reach the cut off for dominancy. If a dominant clone is detected by immunosequencing in such biopsies, it is likely to be distinct from the original malignant clone, presumably representing a new reactive clone.

Although all three patients described in these cases had only one dominant clone, it can be challenging when multiple dominant sequences are identified in one patient (13). Theoretically, one malignant T cell can have up to two rearranged TCRβ sequences. When three or more dominant TCRβ sequences are detected, multiple dominant clones are present in the samples. In such instances, each dominant clone identified needs to be tracked over time, especially the clone that is most prevalent. Future incorporation of clinical-grade RNA-based sequencing assays may improve our understanding of which dominant sequences are relevant to the patient’s disease. Regardless, it is best to track all dominant clones identified by immunosequencing to monitor disease.

In our experience, immunosequencing presents a unique tool to aid in diagnosing difficult cases, differentiating disease progression from cutaneous adverse effects, and monitoring response to treatment. The advantage immunosequencing has over PCR-electrophoresis includes a higher sensitivity to detect malignant clones, track their frequencies over time, and to determine the relationship of the new dominant clone(s) to the original malignant clone (9, 10). We propose to utilize immunosequencing for distinguishing cutaneous adverse events from disease progression in patients with MF treated with mogamulizumab or mechlorethamine gel. Limitations of this study include our small sample size at a single center. We recognize that larger studies are needed to further evaluate the utility of immunosequencing and clone tracking in MF.

## Data availability statement

The original contributions presented in the study are included in the article/supplementary material, further inquiries can be directed to the corresponding author.

## Ethics statement

Ethics approval was obtained for the study involving humans in accordance with the local legislation and institutional requirements. Written informed consent was obtained from the participants or the participants’ legal guardians/next of kin in accordance with the national legislation and the institutional requirements. Written informed consent was obtained from the individual(s) for the publication of any potentially identifiable images or data included in this article.

## Author contributions

SB and DJ wrote and prepared the manuscript for publication. ST contributed to editing and preparing the final manuscript. LB, PP, JM, and JL contributed to editing the manuscript. NN contributed to conceptualization, editing, and writing of the manuscript. All authors contributed to the article and approved the submitted version.
